# Prevalence of HPV Genotypes in South Europe: Comparisons between an Italian and a Turkish Unvaccinated Population

**DOI:** 10.1155/2019/8769735

**Published:** 2019-05-19

**Authors:** Maria Teresa Schettino, Pasquale De Franciscis, Antonio Schiattarella, Viviana La Manna, Alessandra Della Gala, Francesca Caprio, Carolina Tammaro, Franco Pietro Ammaturo, Tolga Guler, Ezgi Hanci Yenigün

**Affiliations:** ^1^Department of Woman, Child, and General and Specialized Surgery, University of Campania “Luigi Vanvitelli”, Largo Madonna Delle Grazie, 1, 80138 Naples, Italy; ^2^Department of Obstetrics and Gynecology, Çamlaraltı Mahallesi, Pamukkale University Medical School, Hastane Yolu, 20070 Denizli, Turkey

## Abstract

The human papilloma virus (HPV) is a DNA virus associated with benign and malignant lesions of skin and mucous membranes and is the most common sexually transmitted viral infection worldwide. We investigated the prevalence of HPV infection and associated risk factors in Italian and Turkish women population attending the gynecology outpatients clinic in Naples (Italy) and Pamukkale (Turkey). Women were enrolled from the Department of Obstetrics and Gynecology of the University of Campania “Luigi Vanvitelli” in Naples (Italy) and of “Pamukkale University” in Denizli (Turkey) between January 2014 and June 2015. A questionnaire that included sociodemographic and sexual behavior characteristics, questions about HPV awareness, vaccine status, and reasons for not wanting to get vaccinated, and HPV-related knowledge was completed for each participant, and cervical cytology samples were collected. The prevalence of HPV infection was higher in the Italian group (52.6% vs 32.6%, *p* < 0.001), while the distribution of genotypes is similar (*p*=0.325). Moreover, the differences in cytological alterations in these patients are significant (*p* < 0.001). The analysis showed a higher prevalence of sexual behavioral characteristics (*p* < 0.001) and better attention to the execution of the screening test in the Italian population (*p* < 0.001). Italian women showed more knowledge and propensity to vaccination compared to Turkish women (*p* < 0.001). Our data highlighted three relevant aspects: the different prevalence of cytological abnormalities, the different distribution of risk factors and, above all, the different attitude of women towards the primary prevention of cervical cancer between an Italian and a Turkish population group.

## 1. Introduction

Human papilloma virus (HPV) is a double-stranded DNA virus belonging to the Papillomaviridae family and is the most common sexually transmitted viral infection worldwide, and it is associated with the occurrence of condylomas and a variety of cancers in both women and men [1, 2]. The HPV-DNA contains sequences that encode E6 and E7, two proteins with oncogenic capacity, that can disrupt the function of two tumor suppressor genes, p53 and pRb, respectively, causing different cellular pathways alterations and uncontrolled cell growth [3]. There are more than 100 HPV types, and the outcome of HPV infection depends on the specific HPV type/s present and can be from asymptomatic infection until high squamous cell malignancies [4]. Low-risk HPV types, such as types 6 and 11, are associated with anogenital warts and mild dysplasia, while high-risk types, such as 16 and 18, are associated with high-grade dysplasia and cancers of the cervix, vulva, vagina, urethra, penis, anus, and oropharynx. Moreover, cervical cancer is the fourth most common type of cancer for women worldwide and the second most common female cancer in women between 15 and 44 years in Europe [5]. Literature evidences that the protection offered by HPV vaccines is enduring and extended vaccination could deliver substantial health economic benefits [6, 7]. Otherwise, monitoring the impact of HPV vaccine effectiveness, in Europe, is challenging because of the variety of factors that need consideration, like the different policies (targeting both sexes and time of vaccination), health system outcomes, and biologic outcomes as infection, risk factors, and sexual behavioral characteristics [8–10]. Vaccination in Italy is recommended and offered to adolescents of both sexes, preferably around 12 years of age: from 9 to 14 years, a bivalent or quadrivalent vaccine was administered in two doses at 0 and 6 months. In later ages, vaccines were administered in three doses at 0, 1, and 6 (bivalent) or 0, 2, and 6 months (quadrivalent vaccine) [11, 12]. The bivalent Cervarix® HPV vaccine (GSK Aspen) targets HPV types 16 and 18, while quadrivalent Gardasil® HPV vaccine (MSD) targets HPV types 6, 11, 16, and 18. A new vaccine will expand coverage against five more oncogenic types (HPV 31, 33, 45, 52, and 58) in addition to the four original types included in quadrivalent vaccine. In Campania, in the south of Italy, the pap test still largely appears as the only screening resource for cervical cancer while the vaccination coverage showed the difficulty of reaching high levels [13, 14]. In Turkey, instead, the National Standards of Cervical Cancer Screening recommends having a smear at least once between the ages of 35 and 40 years with repeat screening every five years, until the age of 65, with two consecutive negative tests. Patients with a cytological or positive anomaly to HPV types 16/18 undergo colposcopy. However, within the same Turkish territory, this screening programme has a highly inhomogeneous distribution because of different and conflicting socioeconomic realities between the territories of western and eastern Turkey. Otherwise, vaccination is only available through private demand [15, 16]. In order to optimize the vaccination programs, it is important to support the beneficial effects of the vaccine and spread awareness about high-risk sexual behavior through the media [17, 18]. Our study aims to compare the Turkish and the Italian population in order to assess the prevalence of HPV infection and the distribution of cytological lesions and the prevalence of sexual behavioral characteristics and environmental risks associated with HPV infection.

## 2. Materials and Methods

We performed a retrospective cohort study of outpatient women who came for gynecological examination, between January 2014 and June 2015, to two referral centers: the Department of Obstetrics and Gynecology of the University of Campania “Luigi Vanvitelli” in Naples (Italy) and of “Pamukkale University” in Denizli (Turkey). The Local Institutional Review Board approved the study. All participants before enrollment signed a full written consent form. The study was conducted in accordance with the principles of Helsinki Declaration. Inclusion criteria were being 25 years of age or older and active sexual history. Patients with autoimmune diseases or any diseases involving the immune system, with HIV infection, previously vaccinated against HPV, with a presumed or confirmed pregnancy, with a diagnosis of any malignancies or with a history of chemotherapy or radiotherapy, and with a history of total uterine or cervical resection, were excluded from the study. The study was performed according to the STROBE (strengthening the reporting of the observational studies in epidemiology) guidelines [19]. Cervical cytology samples were collected, strictly during the nonmenstrual period, by three gynecologists using the Abbott cervi-collect specimen collection kit (Abbott) and transported in ThinPrep® PreservCyt® Solution (HOLOGIC™). These specimens were stored at 15 to 20°C and transported to the laboratory within 24 hours of collection. 15 ml of cervical samples was centrifuged at 2000 rpm for 15 minutes at 4°C for cervical cells concentration. Cell pellet was suspended in 2.5 ml of PBS, and 5 aliquots of 500 *μ*l for each sample were obtained. One aliquot was used for DNA extraction. DNA extraction from a 500 *μ*l cell pellet aliquot of the cervical sample was carried out using the linear array test (Roche Molecular Diagnostics, Milan, Italy), a qualitative in vitro test for the detection of HPV in clinical specimens. The test utilizes amplification of target DNA by polymerase chain reaction and nucleic acid hybridization and detects 37 anogenital HPV-DNA genotypes (6, 11, 16, 18, 26, 31, 33, 35, 39, 40, 42, 45, 51, 52, 53, 54, 55, 56, 58, 59, 61, 62, 64, 66, 67, 68, 69, 70, 71, 72, 73 (MM9), 81, 82 (MM4), 83 (MM7), 84 (MM8), IS39, and CP6108) in cervical cells collected in PreservCyt solution. Three experienced cytology experts performed cervical liquid-based cytology tests and formulated cytological reports according to the Bethesda System [20]. PreservCyt specimens were stored at 15 to 20°C for as long as five weeks, in case the sample had to be retested. Women positive for high-risk HPV were recalled and underwent a colposcopic examination and, if there were an evident lesion, also a targeted biopsy. A targeted biopsy was performed to all those HPV-positive patients with cytological lesions. The cytohistological and viral information obtained was inserted in a specific database. All charts recorded in the database were reviewed carefully by two authors. Data were anonymized before analysis. All patients completed a questionnaire that included sociodemographic and lifestyle information, questions about HPV awareness, vaccine status, and reasons for not wanting to get vaccinated and HPV-related knowledge in order to determine personal risks factors that could increase the susceptibility to HPV. Data were shown as means ± standard deviation (SD) for continuous variables or as number (percentage). Comparisons between the two groups were assessed with Student's *t*-test. Comparison groups were assessed with Pearson's chi-square test and Fisher's exact test for categorical variables and Student's *t*-test for continuous variables. A *p*-value < 0.05 was considered statistically significant. Statistical analysis was made using SPSS for Windows (version 15.0, SPSS, Chicago, IL).

## 3. Results

**Figure 1:**
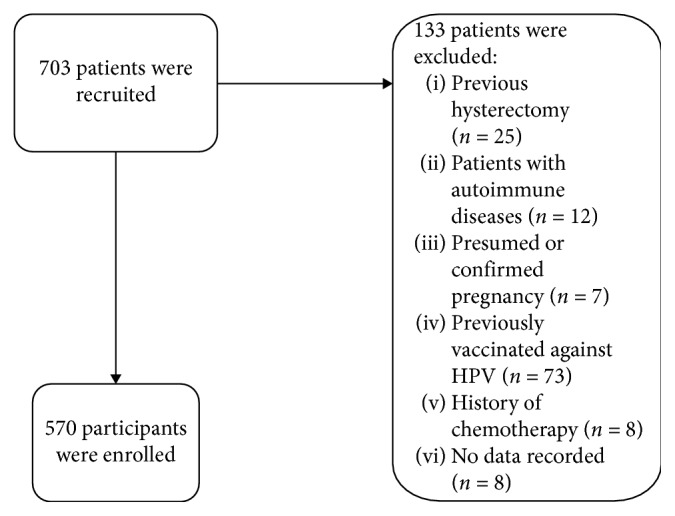
Strengthening the reporting of observational studies in epidemiology (STROBE) statement.

Seven hundred three women were recruited for the study and 570 participants (aged between 25 and 65 years, median 35.6 ± 4.5 years) were enrolled. One hundred thirty-three patients were excluded according to the exclusion criteria as shown in Figure 1.

Patients were divided into two groups according to the country of the enrollment: 300 patients in the Italian group (group A) and 270 patients in the Turkish group (group B). The comparison between the two groups showed that the prevalence of HPV infection was higher in the Italian group than in the Turkey one: 52.6% (*n*=158) vs 32.6% (*n*=88), *p* < 0.05. The distribution of genotypes is similar in the groups of positive HPV patients (*p*=0.325) as shown in Table 1.

Table 2 showed a higher incidence of cytological alterations in group A: the percentage of high-grade lesions (H-SIL) and low-grade lesions (L-SIL), in fact, is higher in group A (20.3% vs 8.0%, *p* < 0.05, and 23.4% vs 6.8%, *p* < 0.05, respectively). Otherwise, group B showed a greater frequency of atypical squamous cells of undeterminated significance (ASCUS) (43.2% vs 25.3%, *p* < 0.05).

Women positive for high-risk HPV were recalled and underwent a colposcopic examination and, if there were an evident lesion, also a targeted biopsy. The results of the biopsies showed a higher incidence of CIN I and CIN II-III in the Italian group and also if not significant (*p*=1.000 and *p*=0.688, respectively) as shown in Table 3.

The questionnaire revealed a greater prevalence of risk factors associated with HPV infection in the Italian group, in particular for low-age first sexual intercourse and the high number of partners in the last three months (*p* < 0.05). Other risk factors like smoking, use of oral contraceptives, and sexually transmitted infections were more prevalent in the Italian group but not statistically significant. Moreover, the Italian group showed greater attention to the execution of the pap test as a screening test (*p* < 0.05), probably related to a higher degree of education (*p* < 0.05). The distribution of risk factors is shown in Table 4.

The two groups showed an overlapping level of knowledge about the screening for cervix cancer, but, surprisingly, group A showed more knowledge about the vaccine and a higher propensity to use it compared to the Turkish group (*p* < 0.05). Moreover, the Turkish group showed a lower willingness to have their children vaccinated against HPV (*p* < 0.05). Data are shown in Table 5.

## 4. Discussion

HPV is the most common viral infection of the reproductive tract, and most people are infected with HPV shortly after the onset of sexual activity [21, 22]. Although most precancerous lesions resolve spontaneously, there is a risk for all women that HPV infection may become chronic and precancerous lesions progressing to invasive cervical cancer in 15 to 20 years [3, 23]. In the era of the vaccine approach as a preventive strategy against cancer associated with HPV infection, epidemiological studies about the distribution of high-risk HPV in different geographical regions are a key tool for several objectives. We need to identify specific risk factors that can be removed with targeted prevention campaigns, to adopt vaccination strategies tailored to every single national reality, to develop vaccines that include prevalent high-risk HPV in different populations, and to identify high-risk genotypes poorly represented in the general population, such as immigrants [24]. Otherwise, our study has some limitations since its results cannot be generalized as it was conducted in two hospitals from two different countries. Moreover, the results of the study are limited to the period when data were collected. However, data showed a similar distribution between the Italian and Turkish groups about the different HPV genotypes but a higher incidence of high-grade cytological lesions in the Italian group. This result could be related to some sexual risk factors that were more frequent in the Italian population, and this point needs to be further studied. As regards the data emerging from the questionnaires, one of the greatest risk factors is represented by smoking due to its potential immunosuppressive effect which can increase the persistence of HPV infection. Literature indicates that the increased risk of cervical cancer is about 2-fold higher among woman smokers compared to nonsmokers, even after appropriate adjustments for sexual habits [25, 26]. However, use of oral contraceptives could promote cervical ectropion that facilitates the exposure of the squamocolumnar junction to potential carcinogens and increased risk of vulvovaginitis and predisposition to dysplasia [27–29]. Alternatively, the use of estrogen and progesterone could maintain infection by increasing cell proliferation and papilloma virus transcription [30, 31]. An innovative aspect of our work is related to recent literature evidence suggesting a role for eating habits and the nutritional status of patients in the progression of cervical carcinoma. In particular, it has been suggested a protective role of folate and vitamins C and E against cervical cancer: they may increase the immune response of the cervix mucosa to HPV infection or act as blockers of free radicals and oxidants that can cause damage to DNA, proteins, and lipids and inhibit the formation of DNA adducts, produced by tobacco smoke [32–34]. In this regard, it was interesting to observe the different eating habits in the two groups. In the typical diet of the Turkish population, there is a high prevalence of daily consumption of raw vegetables and fruit, rich in vitamins and antioxidants: these factors have been called into question with a protective role against the risk of cervical cancer [35, 36]. Moreover, our data showed a better knowledge about HPV of Italian patients compared to Turkish women and higher willingness to have their children vaccinated against HPV. These data are in agreement with two previous studies that revealed that Turkish women have limited awareness and knowledge about the HPV vaccine and a greater need for knowledge [37, 38]. Furthermore, they are likely to reflect the different territorial realities. In Italy, instead, information campaigns for HPV are widespread and the vaccine has been institutionalized since 2007/2008 for all girls from 12 years of age; in 2014, a national coverage of 71% was achieved, with profound differences between the different regional realities ranging between 27% and 86%. In particular, in Campania, the vaccination program reached coverage of 56.1% for a completing vaccination (3 doses) for the 1997 birth cohort on 31/12/2017 [14, 39]. The Italian National Plan of Vaccination Prevention 2017–2019 established the achievement, in girls in the twelfth year of life, of vaccination coverage for a complete cycle of anti-HPV ≥95%; in 2017, the introduction of anti-HPV vaccination for 11-year-old males with the initiation of an active call for the 2006 cohort; and in 2018, the completion of the anti-HPV vaccination in favor of 11-year-old males for the cohort of those born in 2007, with the completion of the recovery of the cohort of those born in 2006 if not achieved in 2017. It should be noted that, in Italy also, the adhesion to screening programs for cervical cancer is very low, with a national average of about 40% [40–42]. Otherwise, in Turkey, there is no similar national vaccine prevention plan for HPV and there is also a lack of a campaign to spread awareness about HPV. Moreover, vaccination is only available through private demand. On the other hand, the level of risk for HPV infection and the prevalence of the infection are much lower than that of Italian women [43, 44]. In order to assess an effective cervical cancer prevention program, it is necessary to implement both strategies: to achieve greater adhesion of the pediatric and adolescent population to the vaccination program, and therefore work to improve communication aspects to families through complete, clear, and transparent information. From this point of view, it would be advisable to make communication more effective by involving other professionals in support of public health facilities such as pediatricians, consultants, general practitioners, gynecologists, and referents of screening programs. Moreover, it should be highlighted the importance to increase adherence to cervical screening programs through the pap smear, which remains an indispensable tool for the cohort of women who got vaccinated.

## 5. Conclusions

Our data underline three relevant epidemiological aspects that could, if confirmed by larger case studies, address the prevention strategies of the cervical cancer and personalize them on the needs of specific realities: the different prevalence of cytological abnormalities, the different distribution of risk factors among the populations under examination and, above all, the different attitude of women towards the primary prevention of cervical cancer. While in Turkey, it is a priority to create a national database of screening and prevention of HPV that is currently lacking and to launch an information campaign and a vaccination plan. In Italy, instead, the level of knowledge and perception regarding the cervical disease, HPV vaccination, and screening for cervical cancer is still not satisfied, and other studies are necessary to give insight regarding knowledge, perception, and acceptance about the HPV vaccine.

## Figures and Tables

**Table 1:** Distribution of genotyping in HPV-positive patients.

	Group A (Italian) (*n*=158)	Group B (Turkish) (*n*=88)	*p* value
Low	41 (26.0%)	23 (26.2%)	1.000
Intermediate	12 (7.5%)	5 (5.6%)	0.793
High	105 (66.5%)	60 (68.2%)	0.887

**Table 2:** Incidence of cytological alterations in HPV-positive patients.

	Group A (Italian) (*n*=158)	Group B (Turkish) (*n*=88)	*p* value
Negative	49 (31%)	37 (42%)	0.094
ASCUS	40 (25.3%)	38 (43.2%)	0.004
L-SIL	37 (23.4%)	6 (6.8%)	0.0008
H-SIL	32 (20.3%)	7 (8.0%)	0.0110

ASCUS: atypical squamous cells of undeterminated significance; L-SIL: low-grade squamous intraepithelial lesions; H-SIL: high-grade squamous intraepithelial lesions.

**Table 3:** Outcome of biopsies.

	Group A (Italian) (*n*=83)	Group B (Turkish) (*n*=13)	*p* value
Negative	56 (67.5%)	11 (84.6%)	0.332
CIN-1	12 (14.5%)	1 (7.7%)	1.000
CIN-2/3	15 (18%)	1 (7.7%)	0.688

CIN: cervical intraepithelial neoplasia.

**Table 4:** Distribution of risk factors.

	Group A (Italian) (*n*=158)	Group B (Turkish) (*n*=88)	*p* value
*Age (mean* *±* *SD)*	32.5 ± 11.4	30.6 ± 9.6	0.187

*Education*			
Low levels of education	23 (14.6%)	28 (31.8%)	<0.001
Upper secondary education	108 (68.4%)	47 (53.4%)	
Graduate education	27 (17.0%)	13 (14.8%)	

*Marital status*			
Married or married-like situation	118 (74.7%)	48 (54.5%)	<0.001
Never married	35 (22.1%)	38 (43.2%)	
Divorced	5 (3.2%)	2 (2.3%)	
Widow	0	0	

*Smoke*			
Ever smoked	82 (52.0%)	35 (40.0%)	0.067
Current or ex-smokers	76 (48.0%)	53 (60.0%)	

*Number of pregnancies*			
None	85 (53.8%)	40 (45.4%)	0.315
From 1 to 3	66 (41.8%)	41 (46.6%)	
More than 3	7 (4.4%)	7 (8.0%)	

*Combined oral contraceptive*			
User	80 (50.6%)	35 (39.8%)	0.101
Not user	78 (49.4%)	53 (60.2%)	

*Age first sexual intercourse (mean* *±* *SD)*	19.8 ± 3.4	26.4 ± 5.3	<0.001

*Number of partners (in the last three months)*			
Only 1	68 (43.3%)	68 (77.0%)	<0.001
From 2 to 3	60 (37.8%)	13 (15.2%)	
From 4 to 9	30 (18.9%)	7 (7.8%)	

*Condom*			
Never used during their sex life	45 (28.5%)	28 (31.9%)	0.582
Usually used during their sex life	113 (71.5%)	60 (68.1%)	

*History of sexually transmitted infection*			
Yes	27 (17.1%)	10 (11.4%)	0.228
No	131 (82.9%)	78 (88.6%)	

*Pap test done in the last 3 years*			
Yes	92 (58.2%)	29 (33.0%)	<0.001
No	66 (41.8%)	59 (67.0%)	

**Table 5:** Knowledge and approach of patients in comparison of HPV and vaccine.

	Group A (Italian) (*n*=158)	Group B (Turkish) (*n*=88)	*p* value
*Knowledge of the motivation of the screening with the pap test*		
Yes	111 (70.2%)	53 (60.2%)	0.109
No	47 (29.8%)	35 (39.8%)	

*HPV knowledge*			
Yes	90 (57.0%)	28 (31.9%)	<0.001
No	68 (43.0%)	60 (68.1%)	

*Knowledge of the anti-HPV vaccine*			
Yes	71 (44.9%)	18 (20.4%)	<0.001
No	87 (55.1%)	70 (79.6%)	

*Knowledge about how the HPV vaccine works*			
Yes	41 (26.0%)	18 (20.4%)	0.333
No	117 (74.0%)	70 (79.6%)	

*Positive propensity for personal use of the vaccine*			
Yes	111 (70.2%)	37 (42.0%)	<0.001
No	47 (29.8%)	51 (58.0%)	

*Positive propensity for the vaccine for children*			
Yes	111 (70.2%)	37 (42.0%)	<0.001
No	47 (29.8%)	51 (58.0%)	

## Data Availability

The data used to support the findings of this study are available from the corresponding author upon request.
